# Adsorptive Removal of Iron Using SiO_2_ Nanoparticles Extracted from Rice Husk Ash

**DOI:** 10.1155/2019/6210240

**Published:** 2019-06-02

**Authors:** Tan Tai Nguyen, Hoa Thai Ma, Pramod Avti, Mohammed J. K. Bashir, Choon Aun Ng, Ling Yong Wong, Hieng Kiat Jun, Quang Minh Ngo, Ngoc Quyen Tran

**Affiliations:** ^1^Department of Materials Science, School of Applied Chemistry, Tra Vinh University, Tra Vinh City 87000, Vietnam; ^2^Department of Active Polymers and Nanomaterials Applications, School of Applied Chemistry, Tra Vinh University, Tra Vinh City 87000, Vietnam; ^3^Department of Biophysics, Postgraduate Institute of Medical Education and Research (PGIMER), Sector-12, Chandigarh 160012, India; ^4^Faculty of Engineering and Green Technology (FEGT), Universiti Tunku Abdul Rahman, Jalan Universiti, Bandar Barat, 31900 Kampar, Perak, Malaysia; ^5^Department of Mechanical and Material Engineering, Lee Kong Chian Faculty of Engineering and Science, Universiti Tunku Abdul Rahman, Sungai Long Campus, Bandar Sg. Long, 43000 Kajang, Malaysia; ^6^Institute of Materials Science, Vietnam Academy of Science and Technology, 18 Hoang Quoc Viet, Cau Giay, Hanoi, Vietnam; ^7^Graduate University of Science and Technology, Vietnam Academy of Science and Technology, 18 Hoang Quoc Viet, Cau Giay, Hanoi, Vietnam; ^8^University of Science and Technology of Hanoi, Vietnam Academy of Science and Technology, 18 Hoang Quoc Viet, Cau Giay, Hanoi, Vietnam; ^9^Graduate University of Science and Technology, Vietnam Academy of Science and Technology, HCM City 70000, Vietnam; ^10^Institute of Applied Materials Science, Vietnam Academy of Science and Technology, HCM City 70000, Vietnam

## Abstract

In this work, SiO_2_ nanoparticles were prepared by the sol-gel method after sodium silicate was extracted from rice husk ash (RHA) under various experimental conditions such as types of acids, NaOH concentration, dissolved time, and temperature and used for removal of Fe^2+^ ions from aqueous solutions. The extracted SiO_2_ was morphologically and chemically characterized and showed a surface area of 78 m^2^/g and uniform pores of 2.71 nm, offering high adsorption capacity for Fe^2+^ ions. The influence of pH, contact time, and amount of adsorbent was studied in order to establish the best conditions for the Fe^2+^ adsorption and removal. Furthermore, the adsorption data were fitted with an exponential shape curve for all the three variable parameters that affect the adsorption process. The best results were obtained for pH 5, 20 min contact time, and 0.5 g adsorbent dose. The loading adsorption capacity was 9 mg of Fe^2+^ ions/g SiO_2_ in the concentration range 0.1–1.0 mgL^−1^. In addition, the synthesized SiO_2_ with the size of around 50 nm can be used for specific heavy metal removal and drug delivery, after modification of the SiO_2_ surface with various functional groups.

## 1. Introduction

In the past few decades, absorptive materials have been developed for removal of heavy metal ions including Hg^2+^, Cu^2+^, Pb^2+^, Zn^2+^, Cd^2+^, and Fe^2+^ from the environmental and biological system due to their toxicity [1–5]. Among all of the heavy metals mentioned, Fe^2+^ ions widely existed in underground water and they are commonly used for household activity in the South of Viet Nam. Long-term drinking water containing high level of Fe^2+^ ions may cause kidney disease, cancer, and anemia along with metabolism disorders [6–12]. Until now, a great deal of effort has been developed for Fe^2+^ ion collection using various techniques, for instance, membrane technology, chemical precipitation coagulation, ion exchange, and electrolytic reduction [13–21]. However, it was known that these methods have several drawbacks such as long time for operation, low capacity for removal, and low thermal and mechanical stability [17, 19, 21]. Among these methods, the adsorption-based technique is a promising technique for removal of Fe^2+^ ions due to its high efficiency, easy operation, cost-effectiveness, and environmental-friendly method. Therefore, several adsorptive materials for removal of Fe^2+^ ions have been widely investigated such as rice husk, activated carbon, fly ash, zeolites, and agricultural by-products [22–27]. It is worth to mention that silica dioxide (SiO_2_) is a promising adsorptive material due to peculiar properties such as porous structure and large surface area. In addition, there are several methods to prepare silica nanoparticles (SNPs) from different agents such as synthesis of nanosilica via the precipitation method with the SNP size of around 50 nm [28, 29] and synthesis of SNPs based on the sol-gel method using rice husk with SNP size of 15 to 90 nm [30–33]. In Viet Nam, the average rice husk produced was around 42 billion tons per year. After burning at high temperature, it became rice husk ash (RHA), which contained a very high amount of silica (approximately 90%). Note that the presence of silica in rice husk had been known since 1938 [34].

In this work, we presented the SNPs efficiency in Fe^2+^ ion adsorption, with SNPs extracted from RHA in brick-kiln industry. We used the sol-gel method for extraction of SNPs under certain controlled conditions such as acid, base, pH, and stirring speed. The physicochemical properties of SNPs were studied for applications of removal of Fe^2+^ ions. The SNPs synthesized could find potential applications including environment for removal of heavy metals and biomedicine for drug delivery.

## 2. Materials and Methods

### 2.1. Agents

Rice husk ash (RHA) was taken from brick-kiln industry. Hydrochloric acid (HCl), nitric acid (HNO_3_), sulfuric acid (H_2_SO_4_), sodium hydroxide (NaOH), iron(II) sulfate, heptahydrate (FeSO_4_·7H_2_O), hydroxylamide (H_2_NO), and 1,10 phenanthroline (C_12_H_8_N_2_) were purchased from Sigma-Aldrich. All agents were diluted in distilled water (DI water).

### 2.2. Methods

#### 2.2.1. Extraction of SiO_2_ Nanoparticles

**Figure 1:**
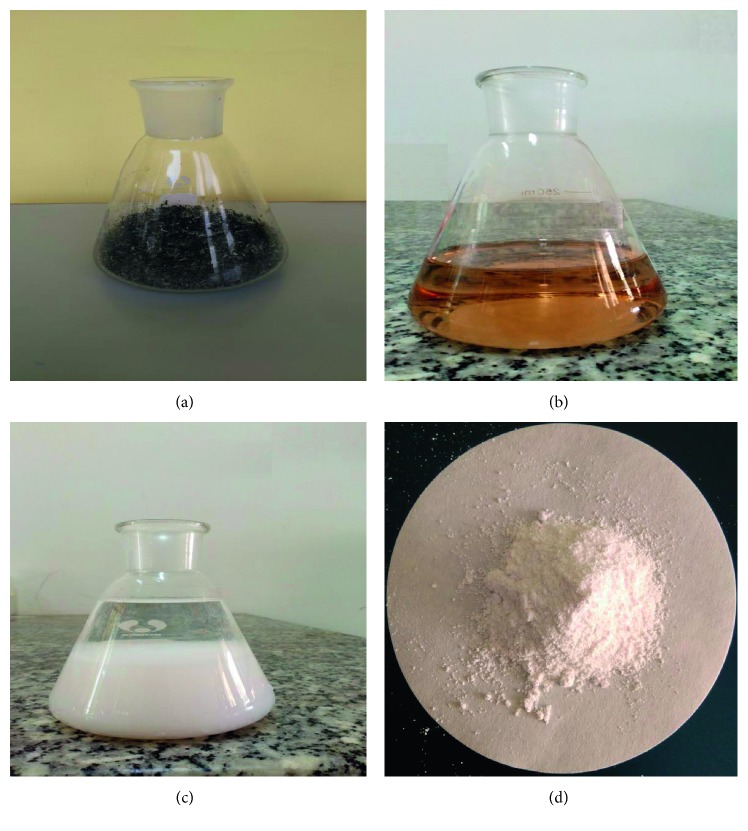
Extraction process of SiO_2_ from rice husk ash. (a) Rice husk ash from brick industry. (b) Rice husk ash diluted in sodium hydroxide after filtering. (c) Rice husk ash solution after precipitation by acid. (d) Extracted SiO_2_ after drying.

SNPs were extracted from RHA based on the sol-gel method.  Figure 1 illustrates the procedure for extraction of SiO_2_ nanoparticles. The extraction process could be briefly described: Firstly, RHA of 2 g was collected from brick-kiln industry and then washed with DI water for removal of dirt. Secondly, RHA was soaked in the sodium hydroxide solvent under stirring at the speed of 400 rpm to generate sodium silicate. The RHA-induced sodium silicate was filtered to remove the nonreactive impurities. Finally, the sodium silicate solution obtained was cooled at room temperature and added to acid under vigorous stirring in order to initiate the hydrolysis-condensation reaction at pH∼7. The gel obtained was then dispersed in ethanol, washed with DI water (three times), and dried at 110°C for 2 h to remove remaining surfactants. The SNPs synthesized were stored in a desiccator for further characterizations. In this work, the effect of acids, NaOH concentration, dissolved time, and temperature was studied.

#### 2.2.2. Fe^2+^ Ion Adsorption Study

The adsorption of Fe^2+^ metal ions from aqueous solution was studied at room temperature. The influence of pH, adsorption time, and mass of adsorbed material was investigated. Consequently, the pH was changed from 3 to 7; adsorption time was set up from 5 to 25 min with increment of 5 min; and mass of SiO_2_ varied between 0.1, 0.5, 1.0, 1.5, and 2.0 g.

For Fe^2+^ ion adsorption measurement, a standard curve was plotted using the concentrations of Fe^2+^ solution in the range of 0.2, 0.4, 0.6, 0.8, and 1.0 ppm. The standard Fe^2+^ solution was generated by mixing FeSO_4_·7H_2_O with 1 ml hydroxylamine hydrochloride, 5 ml phenanthroline, and acetate buffer solution (pH = 3.5). The standard solution generated was kept for 15 min and measured by using a UV-Vis spectrum analyzer. The adsorption capacity (*C*_cap_) and the adsorption efficiency (*E*_eff_) were estimated using the following equation, respectively:

(1) $$\begin{array}{c} C_{\text{cap}}=\frac{C_{\text{in}}-C_{\text{fin}}}{m}V,\\ E_{\text{eff}}=\frac{C_{\text{in}}-C_{\text{fin}}}{C_{\text{in}}}\times 100\%, \end{array}$$

where *C*_in_ and *C*_fin_ are the initial concentration and concentration of equilibrium of Fe^2+^ ions in solution, respectively, *m* is the mass of the adsorbent used, and *V* is the volume of solution.

#### 2.2.3. Physicochemical and Morphological Characterization

Five analytical techniques were used for physicochemical characterization of SiO_2_ extracted and adsorption of Fe^2+^ ions: energy dispersive X-ray spectroscopy (EDS) for the elemental composition of SiO_2_ extracted, transmission electron microscopy (TEM) for ultrastructural analysis; Fourier transform infrared spectroscopy (FT-IR) for characterization of functional groups in the range 4000–500 cm^−1^, ultraviolet-visible spectroscopy (UV-Vis) for determination concentration of solution, surface area measurement by the BET method, and pore size distribution by BJH (Micrometrics ASAP 2010).

## 3. Results and Discussion

**Figure 2:**
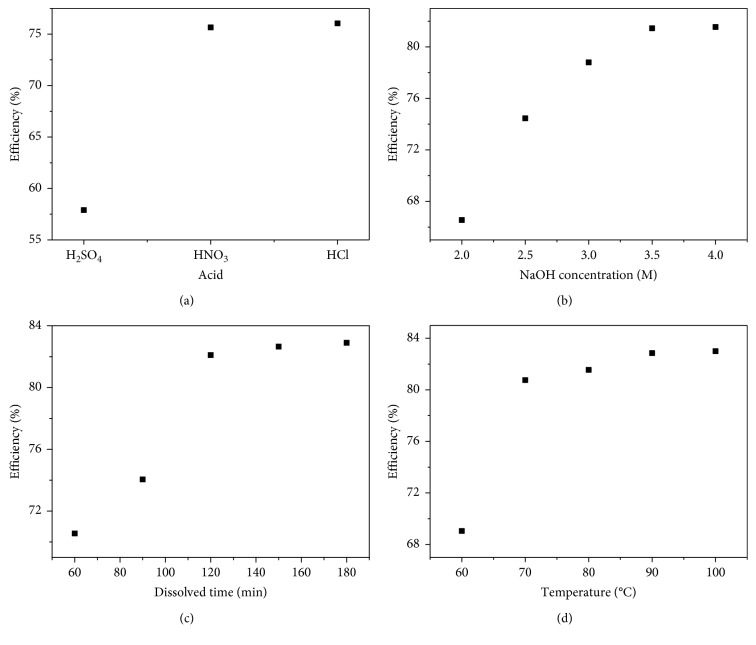
Experimental results of SiO_2_ nanoparticles extraction process. Effect of (a) acids, (b) sodium hydroxide, (c) dissolved time, and (d) temperature.

**Figure 3:**
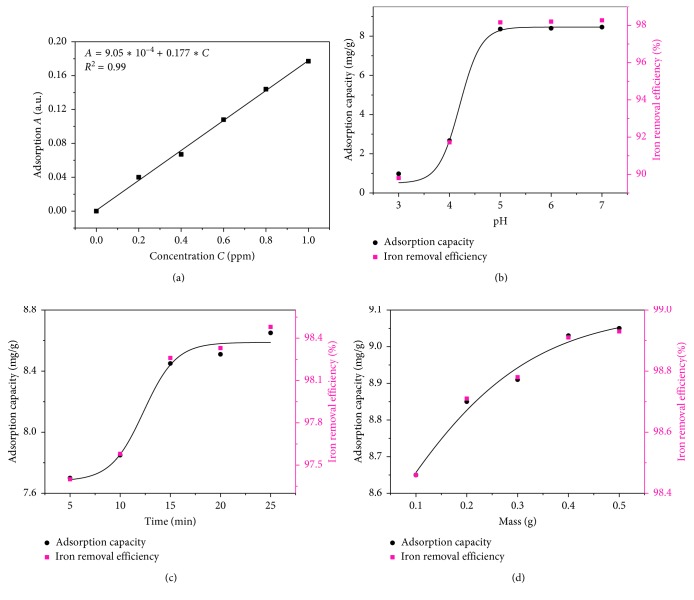
Experimental results of Fe^2+^ adsorption capacity and Fe^2+^ removal efficiency by the SiO_2_ nanoparticles extracted. (a) UV-Vis calibration curve for Fe^2+^ ion adsorption and effect of (b) pH, (c) contact time, and (d) SiO_2_ mass.

In this work, we used 2 g of RHA for synthesis of nanosilica particles. To optimize the conditions for nanosilica synthesis, the effect of dissolved temperature, concentration of sodium hydroxide, concentration of acids, and dissolved time was investigated. The stirring speed of 400 rpm was kept during all the process. Note that each square point in Figures 2 and 3 represents the average value of the three repeated experimental results.

### 3.1. Effect of Acids

The efficiency of SiO_2_ extracted using different acids for neutralization is presented in Figure 2(a). We used three different acids including sulfuric acid, nitric acid, and hydrochloric acid with the same concentration of 3 M for precipitation. The results obtained showed that the extracted efficiency was 58%, 75%, and 76%, respectively. It should be noted that no significant difference was observed using HCl and HNO_3_ for precipitation. The results were better than those of H_2_SO_4_, caused by slow gelation process of silica when sodium silicate reacted with H_2_SO_4_. Thus, HCl was chosen for further study. Herein, concentration of NaOH, temperature, stirring speed, and dissolved time was 3.5 M, 70°C, 400 rpm, and 120 min, respectively. Then, the effect of concentration of HCl was studied in the range from 1 to 5 M. The results showed the highest efficiency of 82%, due to increase in concentration of acids. In addition, the efficiency also depended on the amount of silicate in RHA. So, the optimizing concentration of HCl used was 4 M.

### 3.2. Effect of NaOH

In order to optimize the condition for nanosilica synthesis, an effect of NaOH concentration was investigated. The experiments were performed with NaOH concentration series of 2.0, 2.5, 3.0, 3.5, and 4 M; the dissolved time, concentration of HCl, temperature, and stirring speed were fixed at 120 min, 4 M, 70°C, and 400 rpm, respectively. The results showed that the concentration of NaOH was directly proportional to the efficiency. And, the efficiency of silica synthesized was obtained around 81% at NaOH concentration of 3.5 M as seen in Figure 2(b). This led us believe that the efficiency can be enhanced by controlling the other factors.

### 3.3. Effect of Dissolved Time

Based on the above results, the concentration of NaOH chosen was 3.5 M for further investigating dissolved time. Dissolved time of RHA in NaOH solution was set as a series of 60, 90, 120, 150, and 180 min.  Figure 2(c) shows that the amount of SiO_2_ extracted was gradually increased. Efficiency increased from 70, 82, and 83% for the first, second, and third hours, respectively. The results showed that the amount of SiO_2_ extracted was saturated at 120 min. This was because of the restricted amount of silicate on RHA under burning conditions in brick-kiln industry.

### 3.4. Effect of Temperature

The influence of temperature on the extraction of SiO_2_ from RHA was illustrated in Figure 2(d). This study had been performed using the temperature range from 60°C to 100°C with an interval increment of 10°C. The amount of SiO_2_ extracted had increased with temperature and presented a maximum around 90°C and remained until 100°C. The maximum of extraction mass of SiO_2_ was 1.66 g SiO_2_ in this experiment, corresponding to 83% of efficiency. The efficiency was not higher than that of the other work, due to impurity of RHA in brick-kiln industry under variant conditions in comparison with RHA produced at laboratory with standard conditions [35].

**Figure 4:**
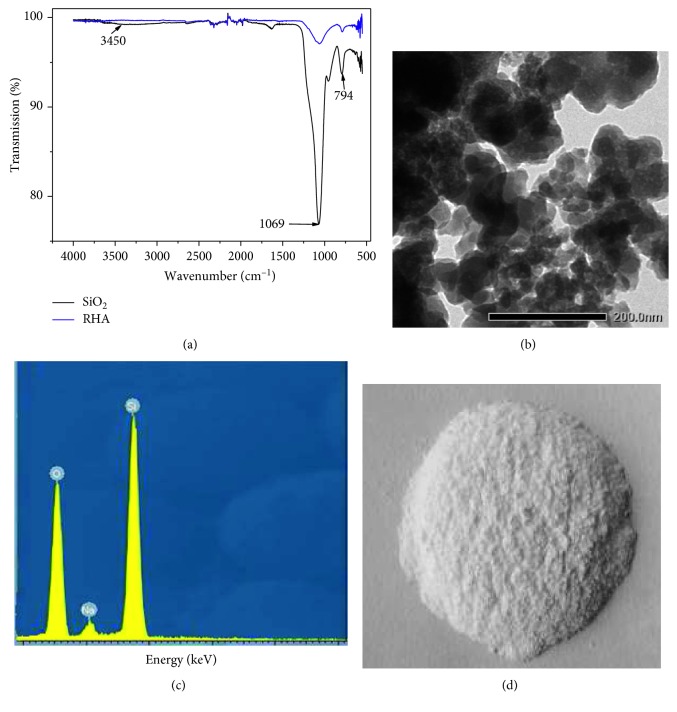
Characterization of SiO_2_ extracted. (a) FT-IR spectrum of the RHA and extracted SiO_2_. (b) TEM image of SiO_2_ nanoparticles. (c) EDS elemental composition analysis of SiO_2_ nanoparticles. (d) SiO_2_ nanoparticles extracted from RHA.

The FT-IR spectrum showed strong adsorption bands at 1069 and 794 cm^−1^ that corresponded to the symmetric and asymmetric Si-O-Si vibration as seen in Figure 4(a), respectively. After extraction of SiO_2_, it was clear that the spectrum differs from the RHA, showing a deeper signal between 1069 and 794 cm^−1^ due to increase in amount of SiO_2_ extracted. In addition, an adsorption peak at 3450 cm^−1^ was associated with the O-H bonds of the silanol groups. Moreover, the main elements consisted of Si, O, and Na with weight concentrations of 23, 75, and 2%, respectively. The small remaining Na was due to unperfect washing. The surface analysis of TEM showed that SiO_2_ had a spherical shape with the diameter of around 50 nm. In addition, BET and BJH analyses of SiO_2_ showed the specific surface area of 78 m^2^/g with a pore size of 2.7 nm. This led us to believe that the extracted SiO_2_ could be used for removal of heavy metal applications.

The adsorption of Fe^2+^ ions by the synthesized SiO_2_ was analyzed.  Figure 3(b) shows the relation of the loading capacity (mg·g^−1^) and adsorption efficiency as a function of pH. At pH < 4, Figure 3(b) presents a low adsorption capacity due to competition between H^+^ ions and Fe^2+^ ions. For pH > 4, concentration of H^+^ decreased, offering the adsorption of Fe^2+^ ions. The adsorption phenomena could be explained by the charge of SiO_2_ dependent on the pH of the surrounding medium. When the pH of the surrounding medium increased, negative charges on the surface of SiO_2_ increased, leading to enhanced electrostatic interaction capacity between SiO_2_ and Fe^2+^ ions as follows:

(2) $$\begin{array}{c} 2\left(-{\text{SiO}}^{-}\right)+{\text{Fe}}^{2+}⟶{\left(-\text{SiO}\right)}_{2}\text{Fe} \end{array}$$

The adsorption of Fe^2+^ ions on SiO_2_ was found between pH 4 and 5. At pH > 5, the small change in adsorption of Fe^2+^ ions resulted from the precipitation of Fe^2+^ and small volume of the pore on SiO_2_ surface. Furthermore, the removal of Fe^2+^ ions was also associated with contact time as seen in Figure 3(c). Results showed that the maximum adsorption efficiency occurred within 20 min with 0.5 g loading mass of SiO_2_ and the maximum adsorption capacity was around 9 mg/g (efficiency of 99%). This result was better than other works using other adsorbents as depicted in Table 1 [36–38]. Note that the higher adsorption capacity would be caused by an increase in the number of active -OH sites on the SNPs surface as presented in Figure 4(a) as well as the surface area and the pore volume of the synthesized SiO_2_.

We fitted the exponential shape curves of the form of *q*=*q*_o_+*a*/[1+exp((*b* − *x*)/*c*)] to the measurement data in Figures 3(b)–3(d) to confirm the characteristic exponential shape of adsorption capacity with variable parameters including pH, time, and mass during adsorption process. Unlike the Langmuir and Freundlich isothermal model, this fitting equation could be used to estimate minimum possible adsorption capacity (*q*_o_) of the adsorbent. As shown in Table 2, the minimum possible adsorption capacity was 7.7, 7.8, and 0.5 mg/g for the case of time change (Figure 3(c)), mass change (Figure 3(c)), and pH change (Figure 3(b)), respectively. We saw that there was large difference in minimum possible adsorption capacity between pH with the other ones. This was due to the fact that there was competitive adsorption occurring between Fe^2+^ ions and H^+^ ions into SNPs at low pH as we already discussed based on equation (2). Moreover, the correlation coefficients (*R*^2^) were both higher than 0.95 indicating that the exponential adsorption data fit well into the model.

The use of the synthesized silica nanoparticles may offer several benefits for drug delivery and adsorption of heavy metals in environment, as mentioned below. Firstly, silica nanoparticles can eliminate the toxicity in comparison with the other particles linked with magnetic nanoparticles or silver nanoparticles when they are introduced into human body for treatment. Secondly, the synthesis process of silica nanoparticles can also be applied for generation of an insulating layer to control electron tunneling between particles, which may be important in charge transfer or magneto-optics. Thirdly, surface modification via functional group immobilization is being pursued with great interest since it can provide unique opportunities to engineer the interfacial of solid substrates while retaining particles' basic geometry. Moreover, the extracted SiO_2_ can be conjugated with various functional groups for specific target detection such as heavy metal ions (Pb^2+^, Cu^2+^, and Cr^6+^) for environmental applications. Finally, the surface area of synthesized SiO_2_ can be increased by using cetyltrimethyl ammonium bromide (CTAB) to enhance capability for drug delivery and heavy metal adsorption.

## 4. Conclusion

We presented the extraction process of SNPs from RHA under different conditions like types of acids, NaOH concentration, dissolved time, and temperature. The results showed that the extraction efficiency was around 83% with purity of 98% and surface area of 78 m^2^/g. Moreover, the Fe^2+^ ion adsorption capacity of the SiO_2_ extracted from RHA was studied under different conditions including pH, contact time, and adsorbent mass. We obtained a maximum loading adsorption capacity of 9 mg Fe^2+^/g SiO_2_ at pH 5 with 20 min of contact time. The adsorption efficiency can be enhanced by modification of SiO_2_ with functional groups. In addition, the synthesized SiO_2_ with the size of around 50 nm can be used for biomedical applications such as drug delivery.

## Figures and Tables

**Table 1:** Comparison of iron adsorption capacity of SiO_2_ extracted from RHA with adsorbent materials in the references.

Adsorbent	Concentration range	Iron adsorption capacity (mg/g)	References
SiO_2_	0.1–1 ppm	9.0	This study
Coir fibers modifying hydrogen peroxide	73–444 mg/L	7.5	[36]
Coir fibers	73–444 mg/L	2.8	[36]
Pine bark waste	55–111 mg/L	2.0	[37]
Cross-linked chitosan	3–9 ppm	64.1	[38]

**Table 2:** Kinetic coefficients in iron adsorption.

	Minimum adsorption capacity, *q*_o_ (mg/g)	Fitting coefficients			*R* ^2^
		*a*	*b*	*c*	
Time	7.7	0.9	12.3	1.6	0.98
Mass	7.8	1.3	1.9	0.1	0.96
pH	0.5	7.9	4.2	0.2	0.99

## Data Availability

The data used to support the findings of this study are available from the corresponding author upon request.
